# Correlation of Serum Intercellular Adhesion Molecule 1 and Transforming Growth Factor Beta 1 With Diabetic Retinopathy in North Indian Patients With Type 2 Diabetes Mellitus

**DOI:** 10.7759/cureus.112938

**Published:** 2026-07-18

**Authors:** Raina Garg, Tejal Srivastava, Abdul Waris, Sheelu Shafiq Siddiqi

**Affiliations:** 1 Institute of Ophthalmology, Jawaharlal Nehru Medical College, Aligarh Muslim University, Aligarh, IND; 2 Institute of Ophthalmology, Jawaharlal Nehru Medical College Hospital (JNMCH), Aligarh Muslim University, Aligarh, IND; 3 Rajiv Gandhi Centre for Diabetes and Endocrinology, Jawaharlal Nehru Medical College Hospital (JNMCH), Aligarh Muslim University, Aligarh, IND

**Keywords:** diabetic retinopathy, endothelial dysfunction, intercellular adhesion molecule 1, transforming growth factor beta 1, type 2 diabetes mellitus

## Abstract

Background: Diabetic retinopathy is a major microvascular complication of diabetes mellitus. Inflammatory and fibrotic pathways, mediated by intercellular adhesion molecule 1 and transforming growth factor beta 1, play crucial roles in its pathogenesis.

Objective: This study aims to investigate the correlation of serum intercellular adhesion molecule 1 and transforming growth factor beta 1 levels with diabetic retinopathy in patients with type 2 diabetes mellitus.

Methods: A hospital-based case-control study was conducted comprising 60 participants, divided into 30 cases with diabetic retinopathy and 30 controls without retinopathy. Fasting blood sugar, postprandial blood sugar, glycated hemoglobin, lipid profile, and renal function parameters were evaluated. Serum intercellular adhesion molecule 1 and transforming growth factor beta 1 levels were measured using enzyme-linked immunosorbent assay. Diabetic retinopathy was graded using the Early Treatment Diabetic Retinopathy Study (ETDRS) classification.

Results: Patients with diabetic retinopathy exhibited significantly higher levels of serum intercellular adhesion molecule 1 (1638.8 ± 839.7 ng/L) and transforming growth factor beta 1 (755.45 ± 237.86 ng/L) compared to controls (315.73 ± 385.80 ng/L and 342.26 ± 138.42 ng/L, respectively) (p < 0.001). Both biomarkers showed significant positive correlations with glycated hemoglobin, fasting blood sugar, postprandial blood sugar, blood urea nitrogen, and serum creatinine. Furthermore, levels of both biomarkers increased progressively with advancing severity of diabetic retinopathy.

Conclusions: Elevated serum intercellular adhesion molecule 1 and transforming growth factor beta 1 levels are significantly associated with the presence and severity of diabetic retinopathy. These biomarkers may serve as valuable noninvasive indicators of endothelial dysfunction, inflammation, and fibrosis in disease progression.

## Introduction

Diabetes mellitus is a critical global public health problem with an alarming increase in prevalence, particularly in developing nations like India [1,2]. The rising rates of type 2 diabetes mellitus have led to a parallel increase in microvascular complications, among which diabetic retinopathy remains a leading cause of preventable blindness in working-age adults [3,4]. Although chronic hyperglycemia and poor glycemic control are established risk factors, the pathogenesis of diabetic retinopathy is complex and multifactorial, involving inflammatory, angiogenic, and fibrotic mechanisms [5,6]. Endothelial activation and leukocyte adhesion play fundamental roles in capillary occlusion and retinal ischemia [7,8]. Intercellular adhesion molecule 1 mediates leukocyte adhesion to the retinal vascular endothelium, leading to increased vascular permeability and microvascular damage [9,10].

Concurrently, persistent oxidative stress activates fibrogenic pathways driven by transforming growth factor beta 1 [11,12]. Transforming growth factor beta 1 promotes extracellular matrix production, basement membrane thickening, and abnormal blood vessel growth, contributing to both early structural alterations and late-stage retinal fibrosis [13,14]. Despite the established roles of these molecules in experimental models, clinical data exploring their combined association with disease severity in the North Indian population remain limited. Therefore, this study was designed to evaluate circulating levels of intercellular adhesion molecule 1 and transforming growth factor beta 1 in patients with type 2 diabetes mellitus and to determine their correlation with the presence and severity of diabetic retinopathy.

## Materials and methods

This hospital-based case-control study was conducted at the Institute of Ophthalmology in collaboration with the Rajiv Gandhi Center for Diabetes and Endocrinology at Jawaharlal Nehru Medical College and Hospital in Aligarh, India. Institutional ethical clearance was obtained prior to the commencement of the study. The study included a total of 60 participants, comprising 30 patients with type 2 diabetes mellitus suffering from diabetic retinopathy as cases and 30 diabetic patients without retinopathy as controls [15]. The majority of participants belonged to the 50-59 years age group (26 participants; 43.3%). This was followed by the 40-49 years and ≥60 years age groups, each comprising 15 participants (25.0%), while only four participants (6.7%) were <40 years of age. Participants were enrolled using a nonprobability consecutive sampling method following the acquisition of informed written consent. Patients with type 1 diabetes mellitus, cardiovascular diseases, diabetic nephropathy, systemic inflammatory disorders, glaucoma, or a history of previous retinal treatments such as laser photocoagulation or intravitreal injections were excluded.

Detailed sociodemographic and clinical data were collected using a predesigned questionnaire. The detailed clinical proforma used for data collection is provided in Appendix 1. Ophthalmic evaluation included the assessment of best-corrected visual acuity, intraocular pressure (IOP) measurement, slit-lamp biomicroscopy, and fundus examination under mydriasis. The severity of diabetic retinopathy was graded according to the Early Treatment Diabetic Retinopathy Study (ETDRS) classification system [16]. 

Furthermore, to allow for a detailed assessment of disease progression, this study utilized a modified binocular 17-step severity scale to generate a single overall diabetic retinopathy severity score for each participant, as detailed in Table 1. 

**Table 1:** Simplified 17-Step DRSS Classification based on the ETDRS Severity Scale ETDRS: Early Treatment Diabetic Retinopathy Study; DRSS: Simplified 17-step Diabetic Retinopathy Severity Scale; DR: diabetic retinopathy; NPDR: nonproliferative diabetic retinopathy; PDR: proliferative diabetic retinopathy; SPC: severe photocoagulation The table outlines clinical descriptions and corresponding scale steps mapped from standard ETDRS severity levels, accounting for single or bilateral eye involvement. Reproduced from Laly et al. [17] under the terms of the Creative Commons Attribution 4.0 International License (CC BY 4.0)

Scale step	Description	Level
1	No DR	10/10
2	Microaneurysms only, one eye	20/<20
3	Microaneurysms only, both eyes	20/20
4	Mild NPDR, one eye	35/<35
5	Mild NPDR, both eyes	35/35
6	Moderate NPDR, one eye	43/<43
7	Moderate NPDR, both eyes	43/43
8	Moderately severe NPDR, one eye	47/<47
9	Moderately severe NPDR, both eyes	47/47
10	Severe or very severe NPDR, one eye	53/<53
11	Severe or very severe NPDR, both eyes	53/53
12	Mild PDR and/or SPC, one eye	60 or 61/<60
13	Mild PDR and/or SPC, both eyes	60 or 61/60 or 61
14	Moderate PDR, one eye	65/<65
15	Moderate PDR, both eyes	65/65
16	High risk PDR, one eye	71+/<71
17+	High risk PDR, both eyes	71+/71+

Biochemical evaluations included fasting blood sugar, postprandial blood sugar, glycated hemoglobin, lipid profile, blood urea nitrogen, and serum creatinine. Venous blood samples were collected under aseptic conditions, and the extracted serum was stored appropriately before analysis. Serum intercellular adhesion molecule 1 and transforming growth factor beta 1 levels were estimated quantitatively using commercially available sandwich enzyme-linked immunosorbent assay kits on a microplate enzyme-linked immunosorbent assay (ELISA) reader. Data analysis was performed using GraphPad Prism (version 10.1.1; GraphPad Software, La Jolla, California, USA), with continuous variables expressed as mean ± standard deviation and categorical variables as frequencies. The independent Student’s t-test, Mann-Whitney U test, and Spearman’s rank correlation were applied as appropriate, with statistical significance defined as a p-value of less than 0.05.

## Results

The study population predominantly consisted of individuals in the 50 to 59 years age group, representing 43.3% of the participants. There was a slight male predominance, with 32 males and 28 females included. Patients with diabetic retinopathy had a significantly higher mean age (56.63 ± 8.73 years) compared to controls (51.26 ± 9.29 years) (p = 0.025) and a significantly longer duration of diabetes (p = 0.028). Based on the ETDRS scale, 13 cases had moderate nonproliferative diabetic retinopathy, 11 had mild nonproliferative disease, four had severe nonproliferative disease, and two had proliferative diabetic retinopathy. The background characteristics of the study participants are summarized in Table 2. 

**Table 2:** Background characteristics of study population

Variable	Category	Frequency (n)	Percentage (%)
Age group	<40 years	4	6.7
	40-49 years	15	25.0
	50-59 years	26	43.3
	>60 years	15	25.0
Sex	Male	32	53.3
	Female	28	46.7

The detailed distribution of diabetic retinopathy based on the ETDRS level of severity among cases is presented in Table 3.

**Table 3:** Distribution of diabetic retinopathy on the basis of ETDRS level of severity among cases (n = 30) ETDRS: Early Treatment Diabetic Retinopathy Study; NPDR: nonproliferative diabetic retinopathy; PDR: proliferative diabetic retinopathy

Stage of DR	ETDRS level	Frequency (n)	Percentage (%)
Mild NPDR	35	11	36.7%
Moderate NPDR	43	13	43.3%
Severe NPDR	53	4	13.3%
PDR	≥61	2	6.7%
Total		30	100%

Glycemic parameters were significantly elevated in the case group, with higher fasting blood sugar (168.11 ± 58.20 mg/dL), postprandial blood sugar (257.18 ± 77.32 mg/dL), and glycated hemoglobin (8.60 ± 1.57%) compared to controls (p < 0.01 for all). Renal and lipid profile parameters, including blood urea nitrogen, serum creatinine, total cholesterol, and triglycerides, were also significantly higher in patients with diabetic retinopathy.

Serum intercellular adhesion molecule 1 levels were markedly elevated in cases (1638.8 ± 839.7 ng/L) compared to controls (315.73 ± 385.80 ng/L) (p < 0.001). Similarly, serum transforming growth factor beta 1 concentrations were significantly higher in cases (755.45 ± 237.86 ng/L) than in controls (342.26 ± 138.42 ng/L) (p < 0.001). Correlation analysis revealed that both intercellular adhesion molecule 1 and transforming growth factor beta 1 exhibited significant positive correlations with glycated hemoglobin, fasting blood sugar, postprandial blood sugar, blood urea nitrogen, and serum creatinine. While transforming growth factor beta 1 showed a significant positive correlation with total cholesterol, its association with triglycerides was not statistically significant. Furthermore, the levels of both biomarkers demonstrated an upward trend with advancing grades of retinopathy severity, showing a pronounced elevation during the transition from nonproliferative to proliferative diabetic retinopathy.

## Discussion

Diabetic retinopathy is driven by a complex interplay of metabolic, inflammatory, and fibrotic mechanisms. In this study, we observed that patients with diabetic retinopathy had significantly elevated serum levels of both intercellular adhesion molecule 1 and transforming growth factor beta 1 compared to diabetic patients without retinopathy.

Regarding intercellular adhesion molecule 1, our findings align with previous literature emphasizing the role of endothelial activation in retinal microvascular injury [18]. Similarly, the elevation of transforming growth factor beta 1 observed in our cases is consistent with existing literature indicating that persistent oxidative stress activates fibrogenic pathways early in the disease process, contributing to both structural alterations and late-stage retinal fibrosis [19].

Mechanistically, intercellular adhesion molecule 1 facilitates leukostasis, leading to capillary occlusion and retinal ischemia, which are hallmark features of diabetic retinopathy [20,21]. Separately, transforming growth factor beta 1 mediates extracellular matrix deposition, basement membrane thickening, and eventual retinal fibrosis [22,23].

Our data demonstrated that both biomarkers correlated positively with poor glycemic control, indicating that chronic hyperglycemia directly stimulates endothelial inflammation and fibrogenic cytokine production [24,25]. Furthermore, the significant association of these biomarkers with renal function parameters such as blood urea nitrogen and serum creatinine suggests a shared pathophysiological mechanism underlying systemic diabetic microangiopathy [26,27].

Pre-existing studies have similarly confirmed that circulating intercellular adhesion molecule 1 levels are strongly associated with the clinical severity of diabetic microvascular complications [28]. The progressive increase in intercellular adhesion molecule 1 and transforming growth factor beta 1 levels from nonproliferative to proliferative stages underscores their potential utility in monitoring disease advancement [28,29]. Specifically, the marked upregulation of these biomarkers in proliferative diabetic retinopathy highlights their active involvement in neovascularization and advanced tissue remodeling [30].

This study is limited by its hospital-based, cross-sectional design and a relatively small sample size from a single center, which may restrict the generalizability of the findings. Additionally, the lack of intraocular fluid analysis prevents a direct comparison between systemic and local retinal biomarker concentrations. Future large-scale longitudinal studies incorporating multiple inflammatory and angiogenic markers are warranted to validate these biomarkers for clinical risk stratification.

## Conclusions

Serum levels of intercellular adhesion molecule 1 and transforming growth factor beta 1 are significantly elevated in patients with type 2 diabetes mellitus suffering from diabetic retinopathy compared to those without retinal involvement. The strong positive correlations between these circulating biomarkers and worsening glycemic, lipid, and renal parameters highlight the interconnected nature of systemic metabolic dysregulation, endothelial inflammation, and progressive fibrotic changes.

The progressive elevation of these biomarkers with advancing grades of retinal disease underscores their potential utility in clinical practice. Assessing these noninvasive serum markers could provide valuable insights for early risk stratification, disease monitoring, and the development of targeted therapeutic strategies aimed at mitigating microvascular complications before the onset of irreversible vision loss.

## Figures and Tables

**Figure 1:**
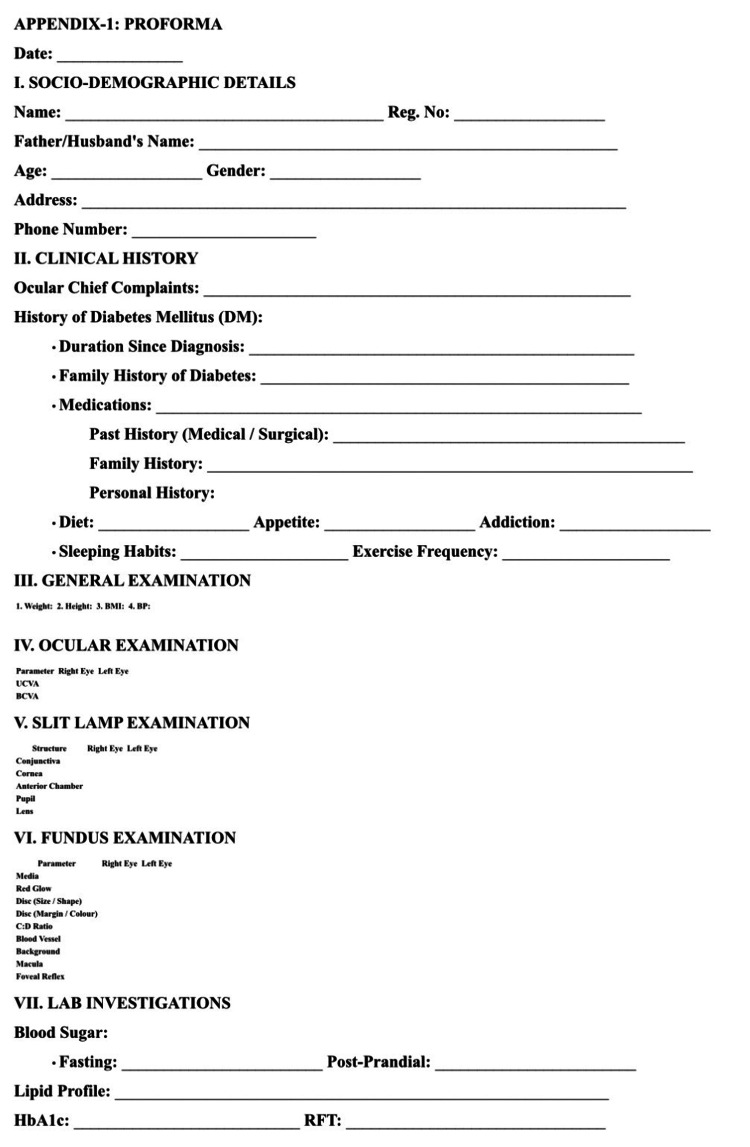
Comprehensive clinical and biochemical data collection proforma

## References

[1] International Diabetes Federation (2021). International Diabetes Federation: IDF Diabetes Atlas. IDF Diabetes Atlas.

[2] Anjana RM, Deepa M, Pradeepa R (2017). Prevalence of diabetes and prediabetes in 15 states of India: results from the ICMR-INDIAB population-based cross-sectional study. Lancet Diabetes Endocrinol.

[3] Arnold SV, Khunti K, Tang F (2022). Impact of micro- and macrovascular complications of type 2 diabetes on quality of life: insights from the DISCOVER prospective cohort study. Endocrinol Diabetes Metab.

[4] Gadkari SS, Maskati QB, Nayak BK (2016). Prevalence of diabetic retinopathy in India: the All India Ophthalmological Society diabetic retinopathy eye screening study 2014. Indian J Ophthalmol.

[5] Marriott HM, Ali F, Read RC, Mitchell TJ, Whyte MK, Dockrell DH (2004). Nitric oxide levels regulate macrophage commitment to apoptosis or necrosis during pneumococcal infection. FASEB J.

[6] Brodsky MC, Aul BJ, Daniels DJ, El-Dairi M (2023). Escape from prism. Surv Ophthalmol.

[7] Ai H, Song HP (2012). Different expression pattern of serum soluble intercellular adhesion molecules-1 and neutrophilic expression of CD18 in patients with diabetic retinopathy. Int J Ophthalmol.

[8] Ceriello A, Falleti E, Bortolotti N (1996). Increased circulating intercellular adhesion molecule-1 levels in type II diabetic patients: the possible role of metabolic control and oxidative stress. Metabolism.

[9] Abdullah RA, Abdulrahman IS (2023). Circulating cell adhesion molecules level in type 2 diabetes mellitus and its correlation with glycemic control and metabolic syndrome: a case-control study. Med J Babylon.

[10] Adamiec-Mroczek J, Oficjalska-Młyńczak J (2008). Assessment of selected adhesion molecule and proinflammatory cytokine levels in the vitreous body of patients with type 2 diabetes-role of the inflammatory-immune process in the pathogenesis of proliferative diabetic retinopathy. Graefes Arch Clin Exp Ophthalmol.

[11] Al-Dahr MH (2017). Association between adhesive molecules and oxidative stress markers among non-insulin dependent diabetic patients. Adv Res Gastroenterol Hepatol.

[12] Hachana S, Larrivée B (2022). TGF-β superfamily signaling in the eye: implications for ocular pathologies. Cells.

[13] Callan A, Jha S, Valdez L, Baldado L, Tsin A (2024). TGF-β signaling pathways in the development of diabetic retinopathy. Int J Mol Sci.

[14] Hudz A, Serhiyenko V, Kudryl I, Guryanov V, Kovtun M, Ziablitsev S (2025). Relationship of transforming growth factor β1 with diabetic retinopathy in type 2 diabetes. Int J Endocrinol (Ukraine).

[15] American Diabetes Association (2020). 1. Improving care and promoting health in populations: standards of medical care in diabetes-2020. Diabetes Care.

[16] Jain A, Saxena S, Khanna VK, Shukla RK, Meyer CH (2013). Status of serum VEGF and ICAM-1 and its association with external limiting membrane and inner segment-outer segment junction disruption in type 2 diabetes mellitus. Mol Vis.

[17] Lally DR, Pepose JS, Magrath GN, Jayagopal A, Kaiser PK (2024). The evolution of clinical trial endpoints for diabetic retinopathy. Retin Physician.

[18] Basyouni M, Ahmed M, Ismail HA, Esmat I (2012). Potential role of oxidized LDL (oxLDL) and adhesion molecules (VCAM-1, ICAM-1) in type 2 diabetes mellitus patients in Qassim region, KSA. J Investig Biochem.

[19] Bonfiglio V, Platania CB, Lazzara F (2020). TGF-β serum levels in diabetic retinopathy patients and the role of anti-VEGF therapy. Int J Mol Sci.

[20] Ekelund C, Dereke J, Nilsson C, Landin-Olsson M (2024). Are soluble E-selectin, ICAM-1, and VCAM-1 potential predictors for the development of diabetic retinopathy in young adults, 15-34 years of age? A Swedish prospective cohort study. PLoS One.

[21] Fotis L, Agrogiannis G, Vlachos IS (2012). Intercellular adhesion molecule (ICAM)-1 and vascular cell adhesion molecule (VCAM)-1 at the early stages of atherosclerosis in a rat model. In Vivo.

[22] Gómez-Bernal F, Quevedo-Abeledo JC, García-González M (2023). Transforming growth factor beta 1 is associated with subclinical carotid atherosclerosis in patients with systemic lupus erythematosus. Arthritis Res Ther.

[23] Jackson M, Ahmad Y, Bruce IN, Coupes B, Brenchley PE (2006). Activation of transforming growth factor-beta1 and early atherosclerosis in systemic lupus erythematosus. Arthritis Res Ther.

[24] Heier M, Margeirsdottir HD, Brunborg C, Hanssen KF, Dahl-Jørgensen K, Seljeflot I (2015). Inflammation in childhood type 1 diabetes; influence of glycemic control. Atherosclerosis.

[25] Hernández C, Burgos R, Cantón A, García-Arumí J, Segura RM, Simó R (2001). Vitreous levels of vascular cell adhesion molecule and vascular endothelial growth factor in patients with proliferative diabetic retinopathy: a case-control study. Diabetes Care.

[26] Badurdeen Z, Alli-Shaik A, Ratnatunga NV (2023). Serum transforming growth factor-beta 1 and creatinine for early diagnosis of CKD of unknown or uncertain etiology phenotypes. Kidney Int Rep.

[27] Jasem AH, Haddad NS, Yaseen NT, Alrufaie MM (2024). Association of TGF-β1 with cystatin-C in patients with diabetic nephropathy. Anaesth Pain Intensive Care.

[28] Ghonaim MM, El-Edel RH (2015). Circulating cell adhesion molecules (sICAM-1 and sVCAM-1) and microangiopathy in diabetes mellitus. Ibnosina J Med Biomed Sci.

[29] Kaneko H, Takayama K, Asami T (2017). Cytokine profiling in the sub-silicone oil fluid after vitrectomy surgeries for refractory retinal diseases. Sci Rep.

[30] Khuu LA, Tayyari F, Sivak JM (2017). Aqueous humour concentrations of TGF-β, PLGF and FGF-1 and total retinal blood flow in patients with early non-proliferative diabetic retinopathy. Acta Ophthalmol.

